# The ovarian reserve is depleted during puberty in a hormonally driven process dependent on the pro-apoptotic protein BMF

**DOI:** 10.1038/cddis.2017.361

**Published:** 2017-08-03

**Authors:** Seng H Liew, Quynh-Nhu Nguyen, Andreas Strasser, Jock K Findlay, Karla J Hutt

**Affiliations:** 1 Development and Stem Cells Program, Monash Biomedicine Discovery Institute and Department of Anatomy and Developmental Biology, Monash University, Clayton, VIC 3800, Australia; 2 The Walter and Eliza Hall Institute of Medical Research, Parkville, VIC 3052, Australia; 3 The Department of Medical Biology, The University of Melbourne, Parkville, VIC 3052, Australia; 4 Centre for Reproductive Health, Hudson Institute of Medical Research, Clayton, VIC 3168, Australia; 5 Department of Molecular and Translational Sciences, Monash University, Clayton, VIC 3168, Australia

## Abstract

In females, germ cells are maintained in ovarian structures called primordial follicles. The number of primordial follicles in the ovarian reserve is a critical determinant of the length of the fertile lifespan. Despite this significance, knowledge of the precise physiological mechanisms that regulate primordial follicle number is lacking. In this study we show that a wave of primordial follicle depletion occurs during the transition to adulthood in mice. We demonstrate that this sudden and dramatic loss of primordial follicles is hormonally triggered and identify the pro-apoptotic BH3-only protein, BCL-2 modifying factor (BMF), as essential for this process, implicating the intrinsic apoptotic pathway as a key mechanism. The elimination of primordial follicles during puberty is not only a striking developmental event, it is also physiologically important because it ultimately reduces the availability of primordial follicles and determines the duration of fertility. Collectively, these findings show that puberty is a critical developmental window for the regulation of the size of ovarian reserve, impacting on female fertility and reproductive longevity.

The ovarian reserve of non-growing primordial follicles represents the entire stockpile of oocytes available to females to support fertility throughout reproductive life.^1^ Most primordial follicles exist in a dormant state, but a few at a time become activated to begin the process of folliculogenesis. Folliculogenesis ultimately terminates in follicular atresia, or less frequently, culminates in the ovulation of a mature oocyte.^2^ The continual entry of primordial follicles into the growing follicle pool leads to the gradual depletion of the ovarian reserve as females age, with infertility occurring when follicle numbers fall below a critical threshold. The pool of primordial follicles can become prematurely depleted by agents that induce genotoxic stress (e.g. many anti-cancer drugs and *γ*-irradiation), leading to oocyte apoptosis and follicle atresia.^3, 4^ However, the extent to which developmentally regulated apoptosis, as opposed to apoptosis induced as a consequence of exposure to specific exogenous stimuli, directly contributes to the normal postnatal decline of primordial follicles is less clear, and the factors that influence the timing of infertility associated with maternal ageing remain poorly characterised.

A number of studies have documented the numeric decline in primordial follicles after birth.^1, 5, 6, 7, 8, 9, 10^ One such study in mice showed that the initial ovarian reserve of primordial follicles is depleted by more than two-thirds between postnatal day (PN) 6 and 45.^8^ After PN45, when mice are considered sexually mature, primordial follicles were lost from the ovarian reserve much more gradually.^8^ Based on these data, and assuming a constant rate of loss, mathematical modelling studies estimated that around 80 primordial follicles are activated each day to become primary follicles, and ~150 primordial follicles undergo atresia each day in the juvenile ovary.^11^ Thus, there is evidence to suggest that apoptosis leading to follicular atresia may contribute significantly to the reduction in the size of the ovarian reserve postnatally, at least in very young mice. However, the concept that large numbers of follicles might be lost during important developmental events postnatally has not been investigated. In particular, primordial follicle numbers and dynamics during puberty in mice have not been studied and the molecular mechanisms underlying postnatal primordial follicle loss have remained elusive.

BCL-2 modifying factor (BMF) is a pro-apoptotic BH3-only protein that functions as an initiator of the intrinsic apoptosis pathway.^12, 13^ The intrinsic apoptosis pathway is triggered by developmental cues and a variety of cell stressors and is regulated by the interplay between three sub-groups of proteins belonging to the BCL-2 family.^14, 15^ The BCL-2 family of proteins are divided into these sub-groups based on their structure and function:^1^ the pro-apoptotic BCL-2 homology domain 3 only containing proteins, known as BH3-only proteins (BIM, PUMA, BID, BAD, BMF, BIK, HRK and NOXA),^2^ the multi BH domain BCL-2-like pro-survival proteins (BCL-2, BCL-XL, MCL-1, A1 and BCL-W) and^3^ the multi-BH domain pro-apoptotic proteins, BAX, BAK and possibly BOK, that unleash the downstream cell demolition events.^16, 17^ The BH3-only proteins initiate apoptosis by binding and neutralising pro-survival BCL-2 family members, which relieves the inhibition of pro-apoptotic BAX and BAK.^15^ Activation of pro-apoptotic BAX/BAK leads to the release of cytochrome c and other apoptogenic factors from the mitochondria, resulting in the formation of the apoptosome, which promotes activation of caspase-9. The effector caspases (e.g. caspase-3, -7) are then activated along with other effector proteins. This ultimately leads to cell demolition with the morphological characteristics associated with apoptosis, including membrane blebbing, chromatin condensation, nuclear fragmentation and engulfment of the apoptotic cell.^18^

We recently reported that ovaries from juvenile (PN20) *Bmf*^*−/−*^ and wild-type (WT) mice had similar numbers of primordial follicles, but while primordial follicle numbers fell considerably in ovaries WT mice by PN100, significantly fewer primordial follicles were lost in the BMF-deficient females during this period.^5^ Furthermore, primordial follicle numbers remained elevated throughout reproductive life and conferred prolonged fertility in *Bmf*^*−/−*^ females.^5^ These findings suggest that BMF may be a key factor in directly or indirectly mediating primordial follicle loss postnatally. In the present study, we focussed on regulation of the size of the ovarian reserve during the period of time that female mice transition from a juvenile status to sexual maturity, referred to as puberty, about which very little is known. We report the following major findings: (1) we show that a wave of primordial follicle loss occurs during puberty in mice, (2) we use a genetic mouse model to demonstrate that apoptosis is required for the pubertal wave of primordial follicle loss and to identify BMF as the key apoptotic protein involved, and (3) we use mouse models of early gonadotropin exposure and puberty suppression to show that primordial follicle loss is actively triggered by gonadotropins. The latter finding reveals a novel and unexpected role for gonadotropins during puberty.

## Results

### The ovarian reserve of primordial follicles is depleted in a BMF-dependent process in mice between PN40 and PN50

**Figure 1:**
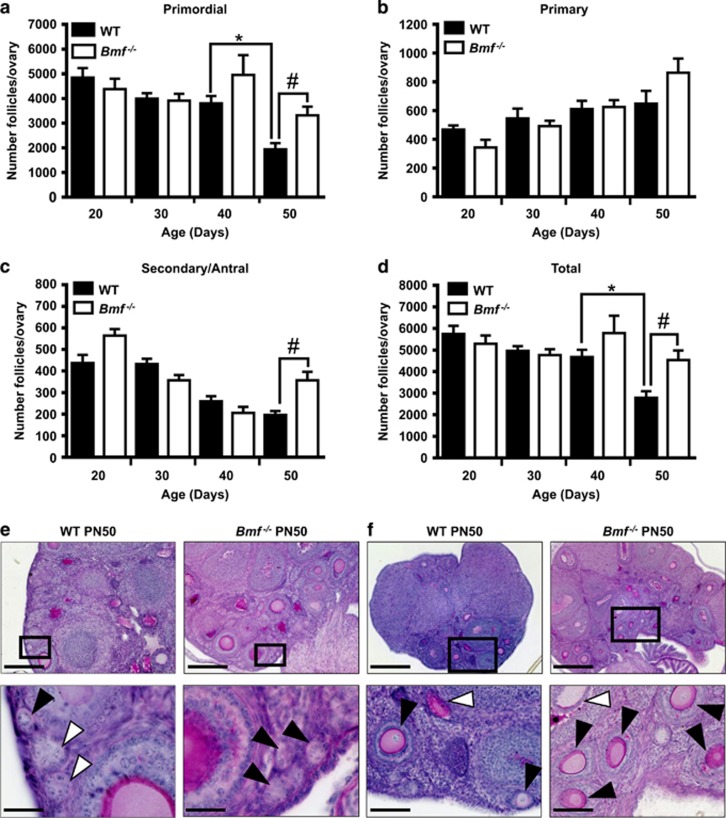
Follicle numbers in ovaries from WT and *Bmf*^−/−^ female mice. Primordial (**a**), primary (**b**), secondary and antral follicles (**c**), and total follicles (**d**) were counted in the ovaries of WT and *Bmf*^−/−^ mice at PN20, 30, 40 and 50 (*n*=6/age/genotype). Data are expressed as mean±S.E.M. *P<0.05 for comparison (two-tailed unpaired *t*-test) of follicle numbers between PN40 and 50 WT female mice. ^#^*P*<0.05 for comparisons (two-tailed unpaired student’s *t*-test) between *Bmf*^−/−^ and WT at PN50. For clarity, only statistical significance of select comparisons are shown. See Supplementary Tables 1 and 2 for statistical significance of all pairwise comparisons within a genotype. (**e**) Representative images of primordial follicles in PAS-stained ovarian sections from WT and *Bmf*^−/−^ mice at PN50. Sections are 20 *μ*M thick enabling primordial follicles to be clearly identified by focussing up and down in the z-axis. Black inset boxes in top images represent area shown below at higher magnification. Black arrow heads indicate primordial follicles. White arrow heads indicate empty follicles. Scale bars: Top images=100 *μ*m, Bottom images=20 *μ*m. (**f**) Representative images of secondary follicles in PAS-stained ovarian sections from WT and *Bmf*^−/−^ mice at PN50. Black inset boxes in top images represent area shown below at higher magnification. Black arrow heads indicate secondary follicles. White arrow heads indicate atretic follicles. Scale bars: Top images=100 *μ*m, Bottom images=20 *μ*m

Follicles were enumerated in ovaries from WT and *Bmf*^−/−^ mice at PN20, 30, 40 and 50, which spans the period of time that female mice transition from juveniles into sexually mature adults (Figures 1a–f). This transitional period is referred to as puberty. The number of primordial follicles was similar in ovaries from WT and *Bmf*^−/−^ mice at PN20, 30 and 40 and remained relatively constant during this time (Figures 1a and e,Supplementary Tables 1 and 2). Strikingly, the number of primordial follicles fell significantly between PN40 and PN50 in ovaries from WT females (Figure 1a), but remained elevated in ovaries from *Bmf*^−/−^ females, pointing to an essential role for BMF in this depletion (Figure 1a,Supplementary Tables 1 and 2). The decrease in primordial follicle numbers in WT ovaries between PN40 and PN50 was not associated with a concomitant increase in primary, secondary or antral follicle numbers, that is, growing follicles (Figures 1a–c), and there was an overall net loss in total follicle numbers (Figure 1d). These data suggest that depletion of the ovarian reserve was not caused by an increased rate of primordial follicle activation and transition into the growing follicle pool. Instead, these observations are consistent with the hypothesis that approximately 50% of the ovarian reserve (~1800 primordial follicles) are directly lost during this 10-day period by BMF-dependent atresia. The dramatic loss of primordial follicles appears to be restricted to this relatively short transitional period, as our previous studies show that depletion of the primordial reserve is much more gradual in adult mice during the remainder of reproductive life.^5^ Interestingly, significantly more secondary and antral follicle were present at PN50 in ovaries from *Bmf*^−/−^ females than WT mice (Figures 1c and f), likely indicative of increased survival in this follicle population. This latter observation is consistent with a critical role of BMF in the secondary and antral follicle atresia that we have previously reported.^5^

### Follicular atresia in adolescent and adult WT and *Bmf*^−/−^ female mice

**Figure 2:**
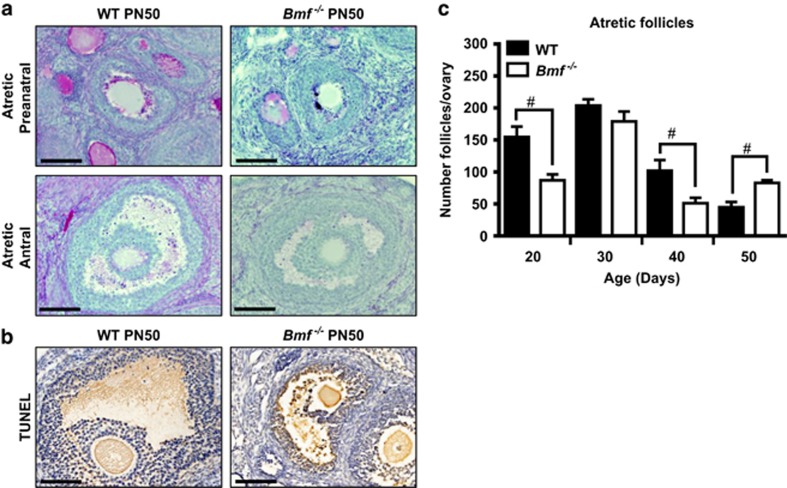
Follicular atresia in ovaries from WT and *Bmf*^−/−^ female mice. The numbers of atretic follicles were determined in ovaries from WT and *Bmf*^−/−^ mice at PN20, 30 40 and 50 (*n*=6/age/genotype). (**a**) Representative images of atretic preantral (secondary) and antral follicles in PAS-stained ovarian sections from WT and *Bmf*^−/−^ mice at PN50. Atretic preantral and antral follicles were characterised by the presence of degrading oocytes and/pyknotic granulosa cells. Scale bars=50 *μ*m. (**b**) Representative images of TUNEL-positive atretic follicles (brown staining) in secondary follicles in ovarian sections from WT and *Bmf*^−/−^ mice at PN50. Scale bars=50 *μ*m. (**c**) Data are expressed as mean±S.E.M. ^#^*P*<0.05 for comparison of *Bmf*^−/−^ *versus* WT females at each age *P*<0.05 (two-tailed unpaired student’s *t*-test). For clarity, only pairwise comparisons between WT and *Bmf*^−/−^ females at each age are shown. See Supplementary Tables 1 and 2 for statistical significance of all pairwise comparisons within a genotype

Follicular atresia was monitored by morphology and apoptotic granulosa cells were confirmed by TUNEL staining (Figures 2a and b). The inability of current markers to detect atretic primordial and primary follicles, even during periods of dramatic follicle loss, has been previously reported^5, 11^ and hence accurate quantification of absolute primordial follicle numbers (as described above) remains the most robust method for detecting their death. Consistent with these earlier studies, we did not find atretic primordial and primary follicles at any age in either genotype of mice. Consequently, our analyses were restricted to secondary and antral follicles. Across the time period analysed, the number of atretic follicles underwent age- and genotype-dependent changes (Figure 2c,Supplementary Tables 1 and 2). Consistent with a role for BMF in mediating follicular atresia, the numbers of atretic follicles were significantly (*P*<0.05) decreased in *Bmf*^−/−^ females at PN20 and PN40 compared to age-matched WT females. However, at PN50 the numbers of atretic follicles were increased in ovaries from *Bmf*^−/−^ females compared to WT females. While unexpected, the increased number of atretic follicles in ovaries from *Bmf*^−/−^ females at PN50 is likely explained by the fact that there were twice as many secondary and antral follicles present in these animals compared to age-matched WT females (Figure 1c). Taking this into account, we then calculated the percentage of the secondary and antral follicle population undergoing atresia and found a slight reduction in atresia in ovaries from *Bmf*^−/−^ females (18.9%) compared to ovaries from WT females (23.0%).

### The pubertal transition in WT and *Bmf*^−/−^ female mice

**Figure 3:**
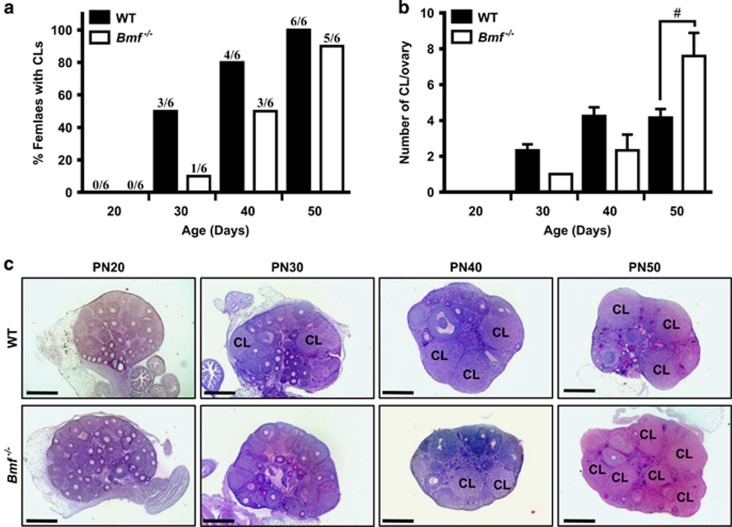
Onset of puberty in WT and *Bmf*^−/−^ female mice. (**a**) The percentages of WT and *Bmf*^−/−^ mice with at least one corpus luteum present in their ovaries were determined at PN20, 30, 40 and 50 (*n*=6/age/genotype). The numbers of mice analysed are shown above each bar. (**b**) The average numbers of corpora lutea present per ovary in WT and *Bmf*^−/−^ mice (only mice with at least one corpora lutea were included in the analysis) were determined at PN20, 30, 40 and 50. Data are expressed as mean±S.E.M. (where *n*=3 or more). ^#^*P*<0.05 for comparison of *Bmf*^−/−^ *versus* WT at PN50 (two-tailed unpaired student’s *t*-test). (**c**) Representative images of PAS-stained ovarian sections from WT and *Bmf*^−/−^ mice at PN20, 30, 40 and 50. The notation ‘CL’ indicate the location of each corpus luteum. Scale bars=200 *μ*m

In order to gain insight into the relationship between the progression to sexual maturity and the observed follicle depletion, we monitored the pubertal transition. We were unable to use traditional methods, such as vaginal opening and vaginal cytology, to compare the onset of puberty in WT and *Bmf*^−/−^ mice because *Bmf*^−/−^ females frequently display delayed vaginal opening or completely imperforate vaginas as a consequence of the requirement for BMF-mediated apoptosis for this process.^19^ Therefore, we used the appearance of corpora lutea as evidence that the mice had begun to ovulate and were progressing through puberty. Corpora lutea were not present in ovaries at PN20 (Figures 3a–c). Corpora lutea were first observed in 3/6 WT females at PN30 and all (6/6) WT females had ovulated by PN50 (Figures 3a–c). Thus, the depletion of primordial follicles in WT females occurred after the first ovulation and coincided with the transition to adulthood. The appearance of corpora lutea was observed at a similar time in *Bmf*^−/−^ mice (Figures 3a–c). Notably, there were significantly more corpora lutea in ovaries from *Bmf*^−/−^ mice compared to WT at PN50 (Figures 3b and c); this may indicate an increased numbers of ovulations or delayed corpora lutea regression, which is in part an apoptotic process.^20^

### Follicle loss during puberty is hormonally triggered rather than age-dependent

**Figure 4:**
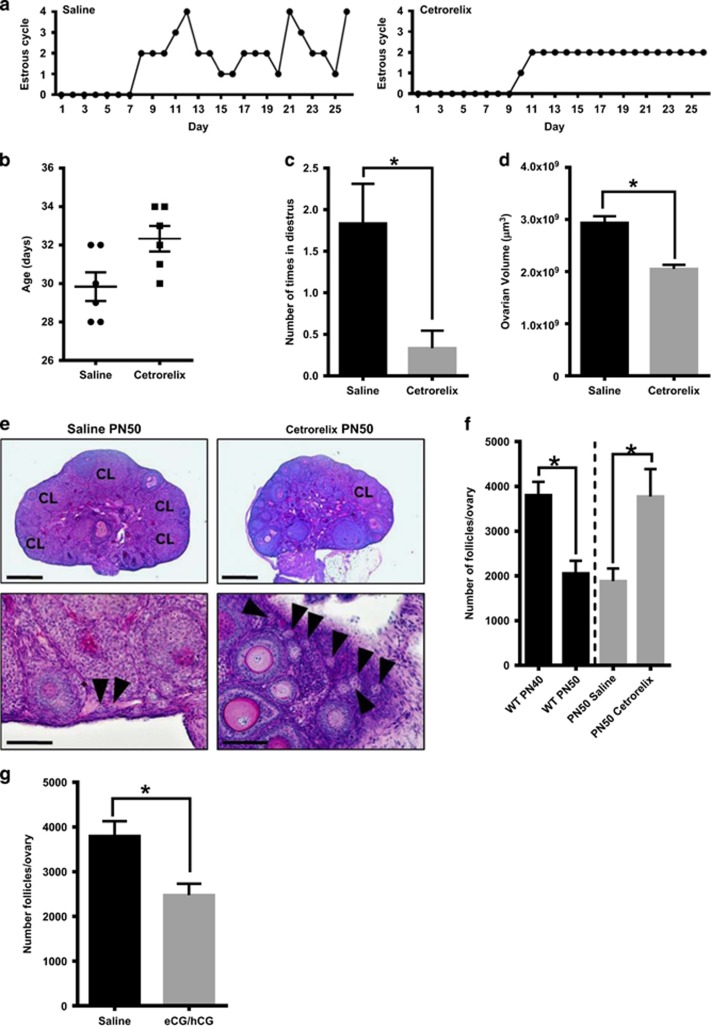
Follicle loss as female mice approach adulthood is hormonally driven. Mice were treated with saline (controls) or Cetrorelix daily beginning at PN25 (Day 1 of the study) and finishing at PN50 (Day 25 of the study) (*n*=6/treatment group) (**a**–**f**). From the time of vaginal opening, mice were smeared daily to monitor the onset of estrous cyclicity. (**a**) Examples of estrus cycling pattern in saline- or Cetrorelix-treated mice. 0= not cycling, vagina not yet opened, 1= metestrus, 2=estrus, 3= proestrus and 4= diestrus. All saline-treated mice displayed onset of estrous cyclicity, whereas mice treated with Cetrorelix either did not cycle or had very disrupted cycles. (**b**) Age at vaginal opening. (**c**) The average number of times mice in saline- and Cetrorelix-treated groups entered diestrus during the 25-day study. Data are expressed as mean±S.E.M. *P<0.05 (two-tailed unpaired student’s *t*-test). (**d**) Ovarian volume. *P<0.05 (two-tailed unpaired student’s *t*-test). (**e**) Representative images of PAS-stained ovarian sections from saline- and Cetrorelix-treated mice. The notation ‘CL’ indicates the location of each corpus luteum. Black arrow heads indicate primordial follicles. Scale bars: Top images=200 *μ*m, Bottom images=40 *μ*m. (**f**) Primordial follicles were counted in ovaries of saline- and Cetrorelix-treated mice at PN50 (grey bars to the right of the dotted vertical line). For comparative purposes, these data are presented alongside the number of primordial follicles in normal WT ovaries at PN40 and PN50 (data from Figure 1a) (black bars to the left of the dotted vertical line). Data are expressed as mean±S.E.M. *P<0.05 (two-tailed unpaired student’s *t*-test). (**g**) Twenty-day-old sexually immature female mice were treated with saline (controls) or equine chorionic gonadotropin (eCG, 5IU), followed 44–48 h later by human chorionic gonadotrophin (hCG, 5IU) (*n*=6/treatment group). Ten days later (PN30), the ovaries harvested and the primordial follicles counted. Data are expressed as mean±S.E.M. *P<0.05 (two-tailed unpaired student’s *t*-test)

Given that the observed depletion of the ovarian reserve in WT females coincided with the transition from a juvenile to adult status, we investigated the possibility that this process was hormonally triggered. We used Cetrorelix, a gonadotropin-releasing hormone (GnRH) antagonist used clinically to delay puberty in girls, to suppress luteinising hormone (LH) secretion in WT mice.^21^ Follicle stimulating hormone (FSH) is also suppressed by Cetrorelix, albeit to a lesser extent.^21^ This treatment resulted in a non-significant delay in vaginal opening (Figure 4b), and effectively disrupted the ability of mice to attain regular estrous cyclicity, enter diestrus and ovulate (Figures 4a and c), and significantly reduced ovarian volume (Figure 4d). These findings are all consistent with lack of cyclic FSH and LH secretion that occurs during the transition into adulthood. Strikingly, Cetrorelix treatment completely prevented the depletion of primordial follicles that was normally observed in WT mice between PN40 and 50 (Figure 4f). Conversely, we found that primordial follicle loss could be triggered abnormally early by treating pre-pubertal mice with hormones (eCG and hCG) that have FSH- and LH-like properties (Figure 4g). Collectively, these data suggest that hormonal status, and not age *per se*, are the key factors in the timing of follicle loss as females transition to sexual maturity.

## Discussion

Rising gonadotropin levels during puberty result in dramatic and dynamic changes in the ovary, including the development of follicles to the Graafian stage and the onset of ovulation. Surprisingly, the regulation of primordial follicle number has not been well studied as the ovary transitions from a pubescent state to a state of full adult function. Although studies of inbred and outbred mouse strains have reported loss of approximately two thirds of the ovarian reserve between the neonatal period and adulthood,^5, 7^ to our knowledge there has not yet been a systematic study of follicle number and loss during puberty. Therefore, the aims of this study were two-fold:^1^ to determine primordial follicle numbers during the transition to adulthood and^2^ to determine if puberty and the associated changing gonadotropic environment impact on the ovarian reserve.

Our results identify puberty as a critical developmental period during which the ovarian reserve undergoes significant changes that ultimately have long-term consequences for reproductive aging and fertile lifespan. Specifically, our data show that there is a pubertal wave of follicle depletion that is gonadotropin-triggered and requires the pro-apoptotic BH3-only protein BMF. That primordial follicle depletion is gonadotropin-regulated is a surprising finding because the survival and development of primordial follicles are often referred to as gonadotropin independent. This understanding is based on the fact that primordial follicles are present and develop up to the late preantral stage in mice lacking functional FSH/LH gonadotropins or their receptors.^22, 23, 24, 25, 26, 27, 28^ However, reports in the literature confirm that FSH and LH can influence primordial follicle number (e.g.^29^). Notably, overexpression of LH in juvenile mice has been shown to trigger depletion of the primordial follicle reserve,^30^ which is in line with the results of our study wherein exogenous hormone stimulation triggered earlier follicle depletion whereas, conversely, suppression of LH and FSH prevented follicle loss. Collectively, these data argue for an important role for LH and/or FSH as direct or indirect physiological triggers for pubertal follicle depletion. It is currently not clear if LH and FSH exert direct actions on primordial follicles as there is controversy regarding the expression of gonadotropin receptors this early in follicle development.^31^ An alternative explanation is that rising levels of LH and/or FSH during puberty indirectly lead to primordial follicle depletion through their influence on the growing follicle pool. Also of note, our data using gene targeted-mice establish BMF as essential for primordial follicle depletion postnatally. BMF is a pro-apoptotic BCL-2 protein family member with documented roles in follicle atresia^5^ and in the regulation of germ cell numbers during fetal ovarian development.^32^ The current results indicate that apoptosis is central to primordial follicle loss during puberty, and they identify BMF as the key apoptotic trigger. Whether BMF acts directly on primordial follicles or regulates apoptosis in growing follicles (or possibly the ovarian stroma) to indirectly mediate primordial follicle depletion, is not known.

While it is not clear why such large numbers of follicles are eliminated at this time of sexual maturation, two recent studies in mice may provide some insight.^33, 34^ These studies suggest that there are two populations of primordial follicles within the ovary that have distinct functional roles.^33, 34^ The primordial follicles that form first during follicular endowment in the fetal ovary are characterised by their location within the medulla and rapid activation at birth.^34^ The majority of these follicles are lost/used before PN60 and primarily contribute to the establishment of functional sexual maturity and endocrine cyclicity.^33^ The cohort of primordial follicles eliminated from the ovary during puberty that we characterised in this study possibly belong to this first population. The second population of primordial follicles is formed slightly later in the fetal ovary, is located in the ovarian cortex and is primarily responsible for adult fertility.^33, 34^ Collectively, these data suggest that there are functional differences in primordial follicles prior to and after puberty and that maturational processes during adolescence, possibly including the loss of abnormal follicles, and increasing follicle developmental competence may be important for the establishment of a mature, fully functional, adult ovary.

Although follicle number in relation to pubertal status has not been specifically studied in girls pre- and post-puberty (and may not be possible due to limited availability of appropriate tissue samples), age-based data suggest that primordial follicle numbers decline by approximately one half during the adolescent/young adult period in humans (ages 13–25 years),^1^ similar to what we have observed in our mouse model. It was also recently reported that primordial follicle populations undergo morphological and functional changes with age and pubertal status in humans:^35^ nearly 20% primordial follicles in pre-pubertal human ovaries were abnormal (defined as follicles with an unusually large oocyte with weakly stained nucleus), whereas abnormal primordial follicles were never observed in adult tissues.^35^ The authors speculated that low quality primordial follicles were eliminated or preferentially utilised prior to the establishment of the ovulation in adulthood. These data suggest that changes in the ovary during the pubertal period extend beyond the cyclic recruitment of antral follicles for ovulation and include changes to the size of the ovarian reserve of primordial follicles.

In summary, we found that approximately 50% of primordial follicles are lost directly from the ovarian reserve immediately prior to adulthood in a process that requires the pro-apoptotic protein BMF and which is gonadotropin triggered. We have identified puberty as an important developmental time point during which the ovarian reserve is significantly depleted and importantly, we have previously shown that this reduction in follicle numbers ultimately reduces the length of the female fertile lifespan.^5^ Our work sheds new light on the developmental mechanisms that regulate the size of the ovarian reserve and determine the number of follicles available to support female fertility. Furthermore, these data provide a new understanding of how the ovarian reserve is regulated over time by showing that rather than a steady loss of primordial follicles postnatally,^6, 7, 8, 9, 10, 11, 36^ a wave of follicle loss is actively triggered at puberty and results in rapid depletion of the follicle pool.

## Materials and methods

### Mice

*Bmf*^−/−^ mice on a C57BL/6 background have been described previously.^13^ C57BL/6 (WT) and Bmf^−/−^ mice were housed in a temperature-controlled high barrier facility (Monash University ARL) with free access to mouse chow and water and a 12 h light-dark cycle. All animal procedures and experiments were performed in accordance with the NHMRC Australian Code of Practice for the Care and Use of Animals and approved by the Monash Animal Research Platform Animal Ethics Committee.

### Assessment of healthy follicle numbers

Unbiased stereology was used to determine follicle number. Stereology is recognised as the best-practice method for the quantification of cells in tissue sections.^37^ Ovaries from WT and *Bmf*^−/−^ mice (PN20, 30, 40, 50, *n*=6/age/genotype) were fixed in Bouin’s solution, processed into hydroxyethyl methacrylate resin (Technovit 7100; Kulzer and Co., Friedrichsdorf, Germany) and then serially sectioned at 20 *μ*m with a Leica RM2165 microtome (Leica Microsystems Nussloch GmbH, Nussloch, Germany). Sections were stained with periodic acid-Schiff and haematoxylin. Methodology for stereological follicle counts has been previously described in detail.^6, 38^ Briefly, we used a × 100 oil immersion objective on an Olympus BX50 microscope (Tokyo, Japan) mounted with an Autoscan stage (Autoscan Systems Pty Ltd, Melbourne, VIC, Australia) which was controlled by the StereoInvestigator stereological system (Version 11.06.02, MBF Bioscience 2015, MicroBrightField, Inc., Williston, VT, USA). Every third section was counted and follicle numbers were determined by multiplying the raw counts of oocytes sampled (Q^−^) by all three sampling fractions (1/f_1_, 1/f_2_ and 1/f_3_).^38^

### Assessment of the numbers of atretic follicles

The numbers of atretic follicles were counted in consecutive 20 *μ*m glycomethacrylate sections throughout the whole ovary using the fractionator/physical disector method as previously described.^38^ Primordial, primary and secondary follicles were classified as atretic if they contained an oocyte that was degenerating (indicated by an irregular of collapsed plasma membrane or a fragmented germinal vesicle), or if granulosa cells were fragmented or had pyknotic condensed nuclei. Antral follicles were considered atretic if more than 10% of their granulosa cells were apoptotic, or if they contained a degenerating oocyte.

### Assessment of puberty: corpora lutea numbers

The numbers of corpora lutea were determined by direct counting of every sixth consecutive 20 *μ*m glycomethacrylate embedded section encompassing the entire ovary. Adjacent sections were evaluated to ensure each corpora lutea was only counted once.

### Suppression of puberty

The gonadotropin-releasing hormone antagonist Cetrorelix (Sigma, Castle Hill, NSW, Australia, Cat #5249) was used to delay puberty in WT mice using daily doses as previously described in rodents.^39, 40, 41^ Injections (0.1 ml) of either saline or the Cetrorelix (0.5 mg/kg body weight/day) were given subcutaneously daily for 25 days (*n*=6 mice/group). Treatment was initiated prior to the onset of puberty, which in female mice is first evident by vaginal opening, occurring at approximately PN28 +/− 2 in our mouse colony. Therefore, treatment was started at PN25 and continued until the conclusion of the experiment at PN50. All animals were monitored daily by visual inspection for vaginal opening. Once vaginal opening was observed, vaginal smears were taken daily to monitor estrus.

### Stimulation with equine chorionic gonadotropin and human chorionic gonadotrophin

PN20 sexually immature female WT mice were injected with saline (controls) or equine chorionic gonadotropin (eCG, 5IU), followed 44–48 h later by injection of human chorionic gonadotrophin (hCG, 5IU) (*n*=6/group). At PN30, the ovaries were harvested and the primordial follicles counted.

### TUNEL staining

The ApopTag Peroxidase *in Situ* Apoptosis Detection Kit (Chemicon International, Melbourne, Australia) was used to detect apoptotic cells in ovarian sections following the manufacturer’s instructions. Apoptotic cells were visualised by the addition of 0.05% DAB chromagen (Sigma, Castle Hill, NSW, Australia) and sections were counterstained using neat Harris haematoxylin. A minimum of three randomly selected slides were evaluated for each animal (*n*=6). Positive controls were performed by exposing slides to DNAseI (according to the kit instructions). On each slide, one section was used for TUNEL analysis, while the other served as a negative control (no addition of TdT).

### Statistical analysis

Data are presented as mean±S.E.M. and statistical analysis of follicle numbers was performed using GraphPad Prism software (GraphPad Software, Inc., La Jolla, CA, USA). Normally distributed data were analysed by student’s *t*-test for pairwise comparisons or one-way ANOVA and the significance was determined by Tukeys *post-hoc* multiple comparison test. Differences were considered significant when *P*<0.05.
