# Supplementary material for: The ovarian reserve is depleted during puberty in a hormonally driven process dependent on the pro-apoptotic protein BMF
Source: Cell Death Dis. 2017 Aug 3;8(8):e2971–. doi: 10.1038/cddis.2017.361 (PMC5596551; doi:10.1038/cddis.2017.361)
Supplement: Supplementary Tables [file cddis2017361x1.docx]

| **Supplemental Table 1. Follicle numbers in WT females** | | | | | | |
| --- | --- | --- | --- | --- | --- | --- |
|  | **Stage of follicular development (mean ± SEM)** | | | | | |
| **Age (PN)** | **Primordial** | **Primary** | **Secondary/Antral** | **Total Growing** | **Total Follicles** | **Atretic** |
| **20** | 4842 ± 387^a^ | 467 ± 29^a^ | 436 ± 39^a^ | 903 ± 60^a^ | 5745 ± 381^a^ | 155± 17^a,b^ |
| **30** | 3988 ± 223^a^ | 542 ± 70^a^ | 432 ± 24^a^ | 978 ± 52^a^ | 4966 ± 215^a^ | 203± 10^a^ |
| **40** | 3805 ± 295^a^ | 612 ± 57^a^ | 260 ± 24^b^ | 871 ± 79^a^ | 4676 ± 336^a^ | 102 ± 17^b^ |
| **50** | 1936 ± 258^b^ | 649 ± 89^a^ | 197 ± 18^b^ | 845 ± 77^a^ | 2781 ± 303^b^ | 45 ± 8^c^ |
| Within a column, values sharing a superscript are not significantly different. Values that do not share a common superscript are significantly different (p<0.05, ANOVA followed by Tukey’s multiple comparison test). | | | | | | |

| **Supplemental Table 2. Follicle numbers in *Bmf^-/-^* females** | | | | | | |
| --- | --- | --- | --- | --- | --- | --- |
|  | **Stage of follicular development (mean ± SEM)** | | | | | |
| **Age (PN)** | **Primordial** | **Primary** | **Secondary/Antral** | **Total Growing** | **Total Follicles** | **Atretic** |
| **20** | 4380 ± 416^a^ | 344 ± 129^a^ | 564 ± 30^a^ | 908 ± 73^a,b^ | 5288 ± 385^a^ | 87 ± 9^a^ |
| **30** | 3912 ± 279^a^ | 492 ± 38^a,b^ | 357 ± 24^b^ | 849 ± 45^a^ | 4761 ± 279^a^ | 179 ± 15^b^ |
| **40** | 4953 ± 803^a^ | 625 ± 48^b,c^ | 205 ± 28^c^ | 831 ± 67^a^ | 5784 ± 809^a^ | 51 ± 9^a^ |
| **50** | 3316 ± 351^a^ | 864 ± 98^c^ | 357 ± 40^b^ | 1221 ± 132^b^ | 4538 ± 440^a^ | 83 ± 4^a^ |
| Within a column, values sharing a superscript are not significantly different. Values that do not share a common superscript are significantly different (p<0.05, ANOVA followed by Tukey’s multiple comparison test). | | | | | | |
